# Supplementary material for: Association of COVID-19 stimulus receipt and spending with family health
Source: PLoS One. 2025 Aug 22;20(8):e0328389. doi: 10.1371/journal.pone.0328389 (PMC12373205; doi:10.1371/journal.pone.0328389)
Supplement: S1 Table — (DOCX) [file pone.0328389.s001.docx]

**S1 Table. Spending variable correlation matrix**

|  | **Savings** | | | **Loans** | | | **Housing** | | | **Household supplies** | | | **Durable goods** | | | **Medical costs** | | |
| --- | --- | --- | --- | --- | --- | --- | --- | --- | --- | --- | --- | --- | --- | --- | --- | --- | --- | --- |
|  | **1** | **2** | **3** | **1** | **2** | **3** | **1** | **2** | **3** | **1** | **2** | **3** | **1** | **2** | **3** | **1** | **2** | **3** |
| **Savings 1** | 1.00 |  |  |  |  |  |  |  |  |  |  |  |  |  |  |  |  |  |
| **Savings 2** | **0.77** | 1.00 |  |  |  |  |  |  |  |  |  |  |  |  |  |  |  |  |
| **Savings 3** | **0.67** | **0.79** | 1.00 |  |  |  |  |  |  |  |  |  |  |  |  |  |  |  |
| **Loans 1** | -0.04 | 0.02 | 0.03 | 1.00 |  |  |  |  |  |  |  |  |  |  |  |  |  |  |
| **Loans 2** | 0.03 | 0.02 | 0.01 | **0.76** | 1.00 |  |  |  |  |  |  |  |  |  |  |  |  |  |
| **Loans 3** | 0.06 | 0.05 | 0.02 | **0.68** | **0.77** | 1.00 |  |  |  |  |  |  |  |  |  |  |  |  |
| **Housing 1** | -0.02 | 0.01 | 0.00 | 0.23 | 0.28 | 0.32 | 1.00 |  |  |  |  |  |  |  |  |  |  |  |
| **Housing 2** | 0.00 | -0.04 | 0.00 | 0.22 | 0.22 | 0.30 | **0.72** | 1.00 |  |  |  |  |  |  |  |  |  |  |
| **Housing 3** | -0.01 | -0.02 | -0.02 | 0.26 | 0.24 | 0.25 | **0.71** | **0.80** | 1.00 |  |  |  |  |  |  |  |  |  |
| **Household supplies 1** | -0.14 | -0.07 | -0.05 | 0.12 | 0.17 | 0.17 | 0.35 | 0.34 | 0.37 | 1.00 |  |  |  |  |  |  |  |  |
| **Household supplies 2** | -0.15 | -0.10 | -0.09 | 0.16 | 0.11 | 0.13 | 0.45 | 0.38 | 0.42 | **0.65** | 1.00 |  |  |  |  |  |  |  |
| **Household supplies 3** | -0.12 | -0.08 | -0.12 | 0.17 | 0.19 | 0.17 | 0.45 | 0.39 | 0.36 | **0.62** | **0.75** | 1.00 |  |  |  |  |  |  |
| **Durable goods 1** | 0.09 | 0.08 | 0.12 | 0.20 | 0.18 | 0.21 | 0.35 | 0.28 | 0.32 | 0.22 | 0.31 | 0.25 | 1.00 |  |  |  |  |  |
| **Durable goods 2** | 0.10 | 0.10 | 0.20 | 0.24 | 0.17 | 0.23 | 0.39 | 0.37 | 0.34 | 0.27 | 0.36 | 0.28 | **0.56** | 1.00 |  |  |  |  |
| **Durable goods 3** | 0.08 | 0.12 | 0.06 | 0.27 | 0.27 | 0.21 | 0.36 | 0.32 | 0.29 | 0.27 | 0.32 | 0.25 | **0.53** | **0.64** | 1.00 |  |  |  |
| **Medical costs 1** | 0.20 | 0.25 | 0.22 | 0.41 | 0.41 | 0.44 | 0.42 | 0.41 | 0.40 | 0.36 | 0.40 | 0.40 | 0.36 | 0.41 | 0.42 | 1.00 |  |  |
| **Medical costs 2** | 0.16 | 0.13 | 0.14 | 0.34 | 0.37 | 0.40 | 0.46 | 0.39 | 0.39 | 0.33 | 0.38 | 0.40 | 0.39 | 0.38 | 0.39 | **0.75** | 1.00 |  |
| **Medical costs 3** | 0.15 | 0.15 | 0.11 | 0.32 | 0.35 | 0.40 | 0.46 | 0.37 | 0.35 | 0.33 | 0.42 | 0.45 | 0.37 | 0.40 | 0.37 | **0.67** | **0.74** | 1.00 |

*Note.* Correlations above 0.50 in bold.
