# Supplementary material for: Association of COVID-19 stimulus receipt and spending with family health
Source: PLoS One. 2025 Aug 22;20(8):e0328389. doi: 10.1371/journal.pone.0328389 (PMC12373205; doi:10.1371/journal.pone.0328389)
Supplement: S2 Tables — (DOCX) [file pone.0328389.s002.docx]

**S2 Table A. Associations of six spending types with mean FHS composite score**

| **FHS Composite** | | | | | |
| --- | --- | --- | --- | --- | --- |
|  | Mean | 95% CI | χ² | P-value |  |
| **Loans** |  |  |  |  |  |
| Mean score | 91.76 | (89.49, 94.02) |  |  |  |
| Mean score at 1 SD above the mean | 91.66 | (88.23, 95.09) |  |  |  |
| Mean difference 1 (1 SD – mean) | -0.10 | (-2.59, 2.40) | 0.01 | 0.939 |  |
| Mean difference 2 within low-income group | 2.98 | (-1.03, 7.00) | 2.12 | 0.145 |  |
| Mean difference 3 within mid-to-high-income group | -1.75 | (-4.76, 1.25) | 1.31 | 0.253 |  |
| Interaction (mean difference 2 – mean difference 3) | 4.74 | (-0.12, 9.59) | 3.66 | 0.056 |  |
| **Savings** |  |  |  |  |  |
| Mean score | 91.77 | (89.50, 94.04) |  |  |  |
| Mean score at 1 SD above the mean | 91.51 | (88.28, 94.73) |  |  |  |
| Mean difference 1 (1 SD – mean) | -0.26 | (-2.66, 2.13) | 0.05 | 0.829 |  |
| Mean difference 2 within low-income group | -2.00 | (-5.69, 1.69) | 1.13 | 0.288 |  |
| Mean difference 3 within mid-to-high-income group | 0.85 | (-2.14, 3.85) | 0.31 | 0.577 |  |
| Interaction (mean difference 2 – mean difference 3) | -2.85 | (-7.48, 1.77) | 1.46 | 0.227 |  |
| **Housing** |  |  |  |  |  |
| Mean score | 91.58 | (89.37, 93.79) |  |  |  |
| Mean score at 1 SD above the mean | 86.89 | (83.60, 90.18) |  |  |  |
| Mean difference 1 (1 SD – mean) | -4.69 | (-7.04, -2.35) | 15.34 | <.0001* |  |
| Mean difference 2 within low-income group | -2.75 | (-6.21, 0.72) | 2.41 | 0.120 |  |
| Mean difference 3 within mid-to-high-income group | -6.24 | (-9.34, -3.14) | 15.59 | <.0001* |  |
| Interaction (mean difference 2 – mean difference 3) | 3.50 | (-1.09, 8.08) | 2.23 | 0.135 |  |
| **Household supplies** |  |  |  |  |  |
| Mean score | 91.64 | (89.41, 93.87) |  |  |  |
| Mean score at 1 SD above the mean | 88.00 | (84.70, 91.29) |  |  |  |
| Mean difference 1 (1 SD – mean) | -3.64 | (-6.00, -1.29) | 9.23 | 0.002* |  |
| Mean difference 2 within low-income group | -1.73 | (-5.74, 2.29) | 0.71 | 0.399 |  |
| Mean difference 3 within mid-to-high-income group | -4.62 | (-7.49, -1.75) | 9.94 | 0.002* |  |
| Interaction (mean difference 2 – mean difference 3) | 2.89 | (-2.02, 7.81) | 1.33 | 0.249 |  |
| **Durable goods** |  |  |  |  |  |
| Mean score | 91.72 | (89.47, 93.98) |  |  |  |
| Mean score at 1 SD above the mean | 89.85 | (86.51, 93.19) |  |  |  |
| Mean difference 1 (1 SD – mean) | -1.87 | (-4.29, 0.55) | 2.30 | 0.129 |  |
| Mean difference 2 within low-income group | 2.08 | (-1.76, 5.91) | 1.13 | 0.289 |  |
| Mean difference 3 within mid-to-high-income group | -4.30 | (-7.32, -1.28) | 7.79 | 0.005* |  |
| Interaction (mean difference 2 – mean difference 3) | 6.38 | (1.54, 11.21) | 6.67 | 0.010* |  |
| **Medical costs** |  |  |  |  |  |
| Mean score | 91.48 | (89.32, 93.65) |  |  |  |
| Mean score at 1 SD above the mean | 85.05 | (81.77, 88.33) |  |  |  |
| Mean difference 1 (1 SD – mean) | -6.43 | (-8.80, -4.07) | 28.46 | <.0001* |  |
| Mean difference 2 within low-income group | -3.01 | (-6.64, 0.62) | 2.64 | 0.104 |  |
| Mean difference 3 within mid-to-high-income group | -8.65 | (-11.60, -5.70) | 32.96 | <.0001* |  |
| Interaction (mean difference 2 – mean difference 3) | 5.64 | (1.06, 10.22) | 5.82 | 0.016* |  |

**S2 Table B. Associations of six spending types with mean Social-Emotional Health scores**

| **Social-Emotional Health** | | | | | |
| --- | --- | --- | --- | --- | --- |
|  | Mean | 95% CI | χ² | P-value |  |
| **Loans** |  |  |  |  |  |
| Mean score | 40.94 | (39.90, 41.99) |  |  |  |
| Mean score at 1 SD above the mean | 41.22 | (39.64, 42.81) |  |  |  |
| Mean difference 1 (1 SD – mean) | 0.28 | (-0.88, 1.43) | 0.22 | 0.638 |  |
| Mean difference 2 within low-income group | 0.86 | (-1.01, 2.72) | 0.82 | 0.367 |  |
| Mean difference 3 within mid-to-high-income group | -0.04 | (-1.43, 1.36) | 0.00 | 0.959 |  |
| Interaction (mean difference 2 – mean difference 3) | 0.90 | (-1.36, 3.15) | 0.61 | 0.436 |  |
| **Savings** |  |  |  |  |  |
| Mean score | 40.96 | (39.92, 42.01) |  |  |  |
| Mean score at 1 SD above the mean | 40.32 | (38.83, 41.81) |  |  |  |
| Mean difference 1 (1 SD – mean) | -0.64 | (-1.75, 0.46) | 1.30 | 0.254 |  |
| Mean difference 2 within low-income group | -1.99 | (-3.69, -0.30) | 5.32 | 0.021* |  |
| Mean difference 3 within mid-to-high-income group | 0.23 | (-1.15, 1.60) | 0.10 | 0.747 |  |
| Interaction (mean difference 2 – mean difference 3) | -2.22 | (-4.34, -0.10) | 4.20 | 0.041* |  |
| **Housing** |  |  |  |  |  |
| Mean score | 40.89 | (39.85, 41.93) |  |  |  |
| Mean score at 1 SD above the mean | 39.61 | (38.06, 41.15) |  |  |  |
| Mean difference 1 (1 SD – mean) | -1.28 | (-2.39, -0.18) | 5.17 | 0.023* |  |
| Mean difference 2 within low-income group | -0.46 | (-2.09, 1.17) | 0.31 | 0.579 |  |
| Mean difference 3 within mid-to-high-income group | -1.93 | (-3.39, -0.47) | 6.75 | 0.009* |  |
| Interaction (mean difference 2 – mean difference 3) | 1.47 | (-0.69, 3.63) | 1.78 | 0.182 |  |
| **Household supplies** |  |  |  |  |  |
| Mean score | 40.90 | (39.86, 41.94) |  |  |  |
| Mean score at 1 SD above the mean | 39.78 | (38.24, 41.31) |  |  |  |
| Mean difference 1 (1 SD – mean) | -1.12 | (-2.22, -0.03) | 4.02 | 0.045* |  |
| Mean difference 2 within low-income group | -0.73 | (-2.61, 1.14) | 0.59 | 0.443 |  |
| Mean difference 3 within mid-to-high-income group | -1.32 | (-2.66, 0.02) | 3.71 | 0.054 |  |
| Interaction (mean difference 2 – mean difference 3) | 0.58 | (-1.71, 2.88) | 0.25 | 0.618 |  |
| **Durable goods** |  |  |  |  |  |
| Mean score | 40.94 | (39.89, 41.99) |  |  |  |
| Mean score at 1 SD above the mean | 41.08 | (39.52, 42.63) |  |  |  |
| Mean difference 1 (1 SD – mean) | 0.14 | (-0.99, 1.26) | 0.06 | 0.810 |  |
| Mean difference 2 within low-income group | 1.47 | (-0.32, 3.26) | 2.61 | 0.106 |  |
| Mean difference 3 within mid-to-high-income group | -0.68 | (-2.09, 0.73) | 0.90 | 0.343 |  |
| Interaction (mean difference 2 – mean difference 3) | 2.16 | (-0.10, 4.41) | 3.51 | 0.061 |  |
| **Medical costs** |  |  |  |  |  |
| Mean score | 40.84 | (39.82, 41.86) |  |  |  |
| Mean score at 1 SD above the mean | 38.67 | (37.12, 40.22) |  |  |  |
| Mean difference 1 (1 SD – mean) | -2.17 | (-3.29, -1.05) | 14.48 | 0.0001* |  |
| Mean difference 2 within low-income group | -1.83 | (-3.57, -0.10) | 4.29 | 0.038* |  |
| Mean difference 3 within mid-to-high-income group | -2.39 | (-3.80, -0.98) | 11.03 | 0.001* |  |
| Interaction (mean difference 2 – mean difference 3) | 0.56 | (-1.63, 2.74) | 0.25 | 0.618 |  |

**S2 Table C. Associations of six spending types with mean Healthy Lifestyles scores**

| **Healthy Lifestyles** | | | | | |
| --- | --- | --- | --- | --- | --- |
|  | Mean | 95% CI | χ² | P-value |  |
| **Loans** |  |  |  |  |  |
| Mean score | 17.38 | (16.93, 17.83) |  |  |  |
| Mean score at 1 SD above the mean | 18.14 | (17.46, 18.82) |  |  |  |
| Mean difference 1 (1 SD – mean) | 0.76 | (0.26, 1.26) | 9.02 | 0.003* |  |
| Mean difference 2 within low-income group | 1.64 | (0.85, 2.44) | 16.53 | <.0001* |  |
| Mean difference 3 within mid-to-high-income group | 0.29 | (-0.31, 0.88) | 0.89 | 0.346 |  |
| Interaction (mean difference 2 – mean difference 3) | 1.36 | (0.40, 2.32) | 7.72 | 0.006* |  |
| **Savings** |  |  |  |  |  |
| Mean score | 17.35 | (16.90, 17.81) |  |  |  |
| Mean score at 1 SD above the mean | 17.40 | (16.75, 18.06) |  |  |  |
| Mean difference 1 (1 SD – mean) | 0.05 | (-0.43, 0.53) | 0.04 | 0.841 |  |
| Mean difference 2 within low-income group | -0.30 | (-1.04, 0.45) | 0.62 | 0.432 |  |
| Mean difference 3 within mid-to-high-income group | 0.27 | (-0.33, 0.88) | 0.78 | 0.376 |  |
| Interaction (mean difference 2 – mean difference 3) | -0.57 | (-1.51, 0.36) | 1.44 | 0.230 |  |
| **Housing** |  |  |  |  |  |
| Mean score | 17.34 | (16.89, 17.80) |  |  |  |
| Mean score at 1 SD above the mean | 16.92 | (16.24, 17.59) |  |  |  |
| Mean difference 1 (1 SD – mean) | -0.43 | (-0.91, 0.06) | 2.97 | 0.085 |  |
| Mean difference 2 within low-income group | -0.39 | (-1.10, 0.33) | 1.11 | 0.292 |  |
| Mean difference 3 within mid-to-high-income group | -0.46 | (-1.10, 0.18) | 1.95 | 0.162 |  |
| Interaction (mean difference 2 – mean difference 3) | 0.07 | (-0.88, 1.02) | 0.02 | 0.883 |  |
| **Household supplies** |  |  |  |  |  |
| Mean score | 17.36 | (16.90, 17.82) |  |  |  |
| Mean score at 1 SD above the mean | 17.41 | (16.73, 18.08) |  |  |  |
| Mean difference 1 (1 SD – mean) | 0.05 | (-0.43, 0.53) | 0.04 | 0.839 |  |
| Mean difference 2 within low-income group | -0.27 | (-1.10, 0.55) | 0.42 | 0.516 |  |
| Mean difference 3 within mid-to-high-income group | 0.21 | (-0.38, 0.80) | 0.51 | 0.477 |  |
| Interaction (mean difference 2 – mean difference 3) | -0.49 | (-1.50, 0.52) | 0.90 | 0.344 |  |
| **Durable goods** |  |  |  |  |  |
| Mean score | 17.35 | (16.90, 17.81) |  |  |  |
| Mean score at 1 SD above the mean | 17.19 | (16.51, 17.86) |  |  |  |
| Mean difference 1 (1 SD – mean) | -0.17 | (-0.66, 0.32) | 0.44 | 0.507 |  |
| Mean difference 2 within low-income group | 0.05 | (-0.74, 0.83) | 0.01 | 0.907 |  |
| Mean difference 3 within mid-to-high-income group | -0.30 | (-0.91, 0.32) | 0.89 | 0.347 |  |
| Interaction (mean difference 2 – mean difference 3) | 0.34 | (-0.65, 1.33) | 0.46 | 0.497 |  |
| **Medical costs** |  |  |  |  |  |
| Mean score | 17.34 | (16.88, 17.80) |  |  |  |
| Mean score at 1 SD above the mean | 16.99 | (16.30, 17.68) |  |  |  |
| Mean difference 1 (1 SD – mean) | -0.35 | (-0.85, 0.14) | 1.94 | 0.163 |  |
| Mean difference 2 within low-income group | -0.08 | (-0.85, 0.70) | 0.04 | 0.848 |  |
| Mean difference 3 within mid-to-high-income group | -0.54 | (-1.16, 0.09) | 2.79 | 0.095 |  |
| Interaction (mean difference 2 – mean difference 3) | 0.46 | (-0.51, 1.43) | 0.86 | 0.355 |  |

**S2 Table D. Associations of six spending types with mean Health Resources scores**

| **Health Resources** | | | | |  |
| --- | --- | --- | --- | --- | --- |
|  | Mean | 95% CI | χ² | P-value | |
| **Loans** |  |  |  |  | |
| Mean score | 22.79 | (21.83, 23.76) |  |  | |
| Mean score at 1 SD above the mean | 21.58 | (20.12, 23.04) |  |  | |
| Mean difference 1 (1 SD – mean) | -1.22 | (-2.28, -0.15) | 5.03 | 0.025* | |
| Mean difference 2 within low-income group | 0.19 | (-1.52, 1.89) | 0.05 | 0.831 | |
| Mean difference 3 within mid-to-high-income group | -1.97 | (-3.25, -0.69) | 9.12 | 0.003* | |
| Interaction (mean difference 2 – mean difference 3) | 2.16 | (0.09, 4.22) | 4.19 | 0.041* | |
| **Savings** |  |  |  |  | |
| Mean score | 22.83 | (21.85, 23.80) |  |  | |
| Mean score at 1 SD above the mean | 22.98 | (21.60, 24.37) |  |  | |
| Mean difference 1 (1 SD – mean) | 0.16 | (-0.87, 1.18) | 0.09 | 0.767 | |
| Mean difference 2 within low-income group | 0.10 | (-1.49, 1.68) | 0.01 | 0.906 | |
| Mean difference 3 within mid-to-high-income group | 0.19 | (-1.10, 1.48) | 0.09 | 0.769 | |
| Interaction (mean difference 2 – mean difference 3) | -0.10 | (-2.09, 1.89) | 0.01 | 0.924 | |
| **Housing** |  |  |  |  | |
| Mean score | 22.73 | (21.80, 23.67) |  |  | |
| Mean score at 1 SD above the mean | 20.11 | (18.72, 21.49) |  |  | |
| Mean difference 1 (1 SD – mean) | -2.63 | (-3.62, -1.64) | 27.14 | <.0001* | |
| Mean difference 2 within low-income group | -1.82 | (-3.28, -0.36) | 6.00 | 0.014* | |
| Mean difference 3 within mid-to-high-income group | -3.27 | (-4.57, -1.96) | 24.10 | <.0001* | |
| Interaction (mean difference 2 – mean difference 3) | 1.44 | (-0.49, 3.38) | 2.15 | 0.143 | |
| **Household supplies** |  |  |  |  | |
| Mean score | 22.76 | (21.82, 23.70) |  |  | |
| Mean score at 1 SD above the mean | 20.55 | (19.16, 21.95) |  |  | |
| Mean difference 1 (1 SD – mean) | -2.21 | (-3.20, -1.21) | 18.96 | <.0001* | |
| Mean difference 2 within low-income group | -0.74 | (-2.42, 0.95) | 0.73 | 0.392 | |
| Mean difference 3 within mid-to-high-income group | -2.95 | (-4.16, -1.75) | 23.02 | <.0001* | |
| Interaction (mean difference 2 – mean difference 3) | 2.22 | (0.15, 4.28) | 4.43 | 0.035* | |
| **Durable goods** |  |  |  |  | |
| Mean score | 22.80 | (21.85, 23.74) |  |  | |
| Mean score at 1 SD above the mean | 20.82 | (19.42, 22.23) |  |  | |
| Mean difference 1 (1 SD – mean) | -1.97 | (-2.99, -0.95) | 14.43 | 0.0001* | |
| Mean difference 2 within low-income group | 0.39 | (-1.20, 1.99) | 0.24 | 0.627 | |
| Mean difference 3 within mid-to-high-income group | -3.42 | (-4.68, -2.17) | 28.65 | <.0001* | |
| Interaction (mean difference 2 – mean difference 3) | 3.82 | (1.81, 5.83) | 13.87 | 0.0002* | |
| **Medical costs** |  |  |  |  | |
| Mean score | 22.70 | (21.78, 23.61) |  |  | |
| Mean score at 1 SD above the mean | 19.51 | (18.13, 20.90) |  |  | |
| Mean difference 1 (1 SD – mean) | -3.18 | (-4.18, -2.18) | 39.08 | <.0001* | |
| Mean difference 2 within low-income group | -0.60 | (-2.10, 0.90) | 0.62 | 0.430 | |
| Mean difference 3 within mid-to-high-income group | -4.85 | (-6.07, -3.63) | 60.90 | <.0001* | |
| Interaction (mean difference 2 – mean difference 3) | 4.25 | (2.36, 6.14) | 19.41 | <.0001* | |

**S2 Table E: Associations of six spending types with mean Social Support scores**

| **Social Support** | | | | |
| --- | --- | --- | --- | --- |
|  | Mean | 95% CI | χ² | P-value |
| **Loans** |  |  |  |  |
| Mean score | 10.64 | (10.19, 11.08) |  |  |
| Mean score at 1 SD above the mean | 10.72 | (10.04, 11.39) |  |  |
| Mean difference 1 (1 SD – mean) | 0.08 | (-0.41, 0.57) | 0.10 | 0.748 |
| Mean difference 2 within low-income group | 0.29 | (-0.50, 1.09) | 0.52 | 0.47 |
| Mean difference 3 within mid-to-high-income group | -0.03 | (-0.63, 0.56) | 0.01 | 0.91 |
| Interaction (mean difference 2 – mean difference 3) | 0.33 | (-0.63, 1.29) | 0.45 | 0.50 |
| **Savings** |  |  |  |  |
| Mean score | 10.63 | (10.18, 11.07) |  |  |
| Mean score at 1 SD above the mean | 10.80 | (10.17, 11.44) |  |  |
| Mean difference 1 (1 SD – mean) | 0.17 | (-0.30, 0.65) | 0.53 | 0.467 |
| Mean difference 2 within low-income group | 0.20 | (-0.53, 0.92) | 0.28 | 0.597 |
| Mean difference 3 within mid-to-high-income group | 0.16 | (-0.43, 0.75) | 0.28 | 0.594 |
| Interaction (mean difference 2 – mean difference 3) | 0.04 | (-0.88, 0.95) | 0.01 | 0.939 |
| **Housing** |  |  |  |  |
| Mean score | 10.62 | (10.18, 11.07) |  |  |
| Mean score at 1 SD above the mean | 10.26 | (9.60, 10.92) |  |  |
| Mean difference 1 (1 SD – mean) | -0.36 | (-0.83, 0.11) | 2.22 | 0.136 |
| Mean difference 2 within low-income group | -0.08 | (-0.77, 0.62) | 0.04 | 0.833 |
| Mean difference 3 within mid-to-high-income group | -0.59 | (-1.21, 0.04) | 3.37 | 0.067 |
| Interaction (mean difference 2 – mean difference 3) | 0.51 | (-0.42, 1.44) | 1.17 | 0.280 |
| **Household supplies** |  |  |  |  |
| Mean score | 10.62 | (10.18, 11.07) |  |  |
| Mean score at 1 SD above the mean | 10.26 | (9.60, 10.91) |  |  |
| Mean difference 1 (1 SD – mean) | -0.37 | (-0.83, 0.10) | 2.35 | 0.125 |
| Mean difference 2 within low-income group | 0.02 | (-0.78, 0.82) | 0.00 | 0.966 |
| Mean difference 3 within mid-to-high-income group | -0.56 | (-1.13, 0.01) | 3.70 | 0.054 |
| Interaction (mean difference 2 – mean difference 3) | 0.58 | (-0.40, 1.56) | 1.34 | 0.246 |
| **Durable goods** |  |  |  |  |
| Mean score | 10.64 | (10.19, 11.08) |  |  |
| Mean score at 1 SD above the mean | 10.76 | (10.10, 11.42) |  |  |
| Mean difference 1 (1 SD – mean) | 0.13 | (-0.35, 0.60) | 0.27 | 0.605 |
| Mean difference 2 within low-income group | 0.16 | (-0.60, 0.93) | 0.17 | 0.679 |
| Mean difference 3 within mid-to-high-income group | 0.10 | (-0.50, 0.71) | 0.11 | 0.735 |
| Interaction (mean difference 2 – mean difference 3) | 0.06 | (-0.91, 1.02) | 0.01 | 0.907 |
| **Medical costs** |  |  |  |  |
| Mean score | 10.60 | (10.16, 11.04) |  |  |
| Mean score at 1 SD above the mean | 9.88 | (9.21, 10.54) |  |  |
| Mean difference 1 (1 SD – mean) | -0.73 | (-1.21, -0.25) | 8.79 | 0.003* |
| Mean difference 2 within low-income group | -0.50 | (-1.25, 0.24) | 1.73 | 0.188 |
| Mean difference 3 within mid-to-high-income group | -0.87 | (-1.48, -0.27) | 7.99 | 0.005* |
| Interaction (mean difference 2 – mean difference 3) | 0.37 | (-0.57, 1.31) | 0.61 | 0.436 |
