# Supplementary material for: Association of COVID-19 stimulus receipt and spending with family health
Source: PLoS One. 2025 Aug 22;20(8):e0328389. doi: 10.1371/journal.pone.0328389 (PMC12373205; doi:10.1371/journal.pone.0328389)
Supplement: Attachment — Submitted filename: Response to Reviewers.docx [file pone.0328389.s006.docx]

# Response to Reviewers

We would like to thank the reviewers for their suggestions. We have addressed and responded to each of the issues raised below.

**Reviewer #3**

*1. Perhaps I did not see, or my ignorance, but mTurk is unfamiliar to me ...if possible pls. add something from "Amazon Mechanical Turk (MTurk) is a crowdsourcing marketplace that makes it easier for individuals and businesses to outsource their processes and jobs to a distributed workforce who can perform these tasks virtually. This could include anything from conducting simple data validation and research to more subjective tasks like survey participation, content moderation, and more. MTurk enables companies to harness the collective intelligence, skills, and insights from a global workforce to streamline business processes, augment data collection and analysis, and accelerate machine learning development."*

We appreciate the suggestion to elaborate on Amazon mTurk. We have added/revised some sentences in the “Participants and Sampling” paragraph to further explain how mTurk works: "MTurk is a crowdsourcing marketplace that businesses and individuals can use to outsource data collection and processing for various purposes, including research. MTurk users provide a sample more diverse than typical convenience samples, and complete tasks requested by researchers, such as surveys, data validation, and more. The mTurk users receive an incentive, typically financial compensation, for their work."

*2."147 known as active response rate [2432]. The 500-participant sample size would allow sufficient 1" I see 456=n, please fix this minor item.*

We thank the reviewer for identifying this mistake. We have changed the manuscript to read, "The 456-participant sample size..."

*3. The paper defines income categories differently in various sections. For example, it mentions "<$25,000" as low income in one part, but later uses "<$40,000" as the low-income threshold. or perhaps it is on the cut-off, if possible please make it clearer.*

We appreciate the reviewer identifying this discrepancy. The <$25,000 threshold mentioned in the paragraph on “Participants and Sampling” was used to ensure that the sample we gathered would include an adequate number of low income families. In contrast, the <$40,000 threshold mentioned in the paragraph on “Demographics” was used to define a low income category for the data analysis. We have revised both paragraphs to clarify this difference.

*4. The explanation of factor analysis mentions six factors, but Eigenvalues only exceed 1.0 for five factors. The text acknowledges this discrepancy without resolving it, raising doubts about whether the six-factor solution is valid (lines 226-229). A non-zero vector v is an eigenvector of A if Av = λv for some number λ, called the corresponding eigenvalue. The eigenvector with the largest eigenvalue is the direction with most variability, this eigenvector is the first principle component. It may be possible there is some factor instability drift and heteroscedasticity (which is fine), "Agrrawal and Clark. "ETF Betas: A Study of their Estimation Sensitivity to Varying Time Intervals." ETFs and Indexing (2007)", attribute factor/coefficient instability to heterogenous variance discontinuities and note their impact on multiple orthogonal factors for an overall ranking scale. A similar effect could slightly be at play here. Additionally data driven methods to identify spatial patterns by optimizing euclidean distances is utilized in "Heumann et al. Data-Driven Algorithm to Redefine the US Rural Landscape: Affinity Propagation as a Mixed-Data/Mixed-Method Tool. Economic Development Quarterly (2022)."*

We thank the reviewer for suggesting that we clarify our rationale for retaining six factors when the sixth factor had an eigenvalue of 0.9 instead of >1.0. We did not rely solely on the eigenvalue-greater-than-one rule. We have revised the "Spending Patterns Determination" paragraph to explain our evaluation of the principal component analysis in more detail, including our rationale for retaining six factors.

*5. "Figure 1 Legend" section, where the explanation appears without proper figure presentation/title (lines 308-317), an appendix perhaps?*

We thank the reviewer for recognizing this. Per PLOS One submission guidelines, "Figure captions are inserted immediately after the first paragraph in which the figure is cited. Figure files are uploaded separately." Figure 1 is first cited in the “Spending Factor Analysis” paragraph, therefore the Figure 1 legend is provided right after that paragraph, per journal instructions. There is an additional file that is uploaded separately from the manuscript file, labeled Fig 1.

*6. [line 84-86] Most U.S. research points to economic and employment constraints [10-12-14], interruptions to family routines [135], reduced quality of life and family well-being [810, 146], psychological distress [810, 157-179]. A recent large scale study using 2 million non-natural deaths using CDC and NVDRS data establishes a lagged link between finance induced stress and subsequent period suicides. (Sandweiss et al. "Suicides as a response to adverse market sentiment. PLoS One, 2017."*

We appreciate the reviewer sharing this reference. We have now cited it to support the point about psychological distress.

**Reviewer #4**

*Your data sources are complex, it may be difficult for researcher students to provide such data. You can mention an early stage AI knowledge paper that shows that the utilization of automated web-harvesting algorithms can easily provide the researcher with zero-cost machine readable datasets for further analysis.*

We appreciate the reviewer's suggestion of using automated web-harvesting algorithms. However, we do not do that in our paper; we use Amazon mTurk to outsource survey responses from a more diverse population. Thus, it may not be applicable to our paper to make reference to such web-harvesting algorithms. Rather, we have added/revised some sentences in the “Participants and Sampling” paragraph to further explain how mTurk works: "MTurk is a crowdsourcing marketplace that businesses and individuals can use to outsource data collection and processing for various purposes, including research. MTurk users provide a sample more diverse than typical convenience samples, and virtually complete tasks requested by researchers, such as surveys, data validation, and more. The mTurk users receive an incentive, typically financial compensation, for their work."

*Some ambiguity wrt your eigenvector decomposition < 1 seems to exist. PCA modeling could be tightened.*

We thank the reviewer for suggesting that we clarify our rationale for retaining six factors when the sixth factor had an eigenvalue of 0.9 instead of >1.0. We did not rely solely on the eigenvalue-greater-than-one rule. We have revised the "Spending Patterns Determination" paragraph to explain our evaluation of the principal component analysis in more detail, including our rationale for retaining six factors.
